# Vaccines for TB: Lessons from the Past Translating into Future Potentials

**DOI:** 10.1155/2015/916780

**Published:** 2015-06-03

**Authors:** Gee Jun Tye, Min Han Lew, Yee Siew Choong, Theam Soon Lim, Maria Elena Sarmiento, Armando Acosta, Mohd Nor Norazmi

**Affiliations:** ^1^Institute for Research in Molecular Medicine, Universiti Sains Malaysia, 11800 Minden, Penang, Malaysia; ^2^ADAPT Research Cluster, Centre for Research Initiatives, Clinical & Health Sciences, Universiti Sains Malaysia, 16150 Kubang Kerian, Kelantan, Malaysia; ^3^School of Health Sciences, Universiti Sains Malaysia, Health Campus, 16150 Kubang Kerian, Kelantan, Malaysia

## Abstract

Development of vaccines for infectious diseases has come a long way with recent advancements in adjuvant developments and discovery of new antigens that are capable of eliciting strong immunological responses for sterile eradication of disease. Tuberculosis (TB) that kills nearly 2 million of the population every year is also one of the highlights of the recent developments. The availability or not of diagnostic methods for infection has implications for the control of the disease by the health systems but is not related to the immune surveillance, a phenomenon derived from the interaction between the bacteria and their host. Here, we will review the immunology of TB and current vaccine candidates for TB. Current strategies of developing new vaccines against TB will also be reviewed in order to further discuss new insights into immunotherapeutic approaches involving adjuvant and antigens combinations that might be of potential for the control of TB.

## 1. Introduction

T-cell vaccination is the administration of antigenic material to produce T-cell immunity against certain diseases. Antigens used in vaccinations range from live viruses or bacteria to peptides of proteins associated with the disease target. Edward Jenner marked the beginning of modern vaccination by using cowpox to give protection against smallpox in humans in 1796. Injecting the harmless form of a disease organism, Jenner utilized the specificity and memory of the acquired immune response to lay the foundation for modern vaccination strategies [1]. For the control of infectious diseases, the core objectives are prophylaxis and therapy. The initial priority of vaccine development has always been prophylaxis, but the development and evaluation of therapeutic vaccines, mainly for chronic infectious diseases and cancer, are gaining momentum. Therapeutic vaccines are substantially more challenging, as diseases such as cancer or chronic infectious diseases would have coexisted with the immune system for a long time and have finally escaped immune surveillance when symptoms are visible. At this stage, almost by definition, tolerance would have been induced [2, 3]. The exposition to* Mycobacterium tuberculosis* (*M. tb*) produces several outcomes: no infection, latent infection, or active disease [4, 5]. During the duration of the latency period,* M. tb* goes into a dormant state and would be controlled by the host's immunity. However, this control would slowly bring the immune system to a halt via blockade of regulatory T-cells (Treg) conversion to T_H_17 cells, blockade of T_H_2 cells providing stimulation to memory T-cells (T_M_), and blockade of costimulatory ligands to avoid activation of effector T-cells (T_Eff_) [5]. In this context, the immune system loses the capability to contain the reactivation of* M. tb*, resulting in overt clinical disease, represented by nearly 8 million of new cases of TB every year. This situation is aggravated by the growing appearance of multidrug resistant TB (MDR-TB), extensive resistant TB (XDR-TB), and totally resistant TB (TDR-TB). In MDR-TB and XDR-TB, therapy requires alternative, long, expensive, and toxic treatments with low success rates while TDR-TB is untreatable. This dire situation now prompts the need for immunotherapies, with special interest in T-cell based vaccines to reactivate cytotoxic T-cells with the capacity to clear disease [6].

## 2. An Overview of* Mycobacterium tuberculosis* and Immunity

TB is a disease of the respiratory tract that is transmitted through airborne* M. tb*. Infection of* M. tb* begins with the deposition in the alveolar spaces of the lungs upon inhaling air with airborne* M. tb*. Here,* M. tb* will be phagocytosed by alveolar macrophages (AM) and destroyed with the help of complement activation [7–9]. Cells such as dendritic cells also play a part in this process [10–12] but some of the* M. tb* will be able to escape the process of intracellular destruction by the innate immunity, thus leading to multiplication of the infection and destruction of the AM. Cell debris from the destroyed AM is then ingested by other monocytes which would normally differentiate to other macrophages that would be ready to phagocytose other* M. tb* but does not destroy the internalized* M. tb* from the AM cell debris.

Upon development of acquired immunity (T/B-cell responses), early infected cells would undergo cytotoxicity. Other AM which were secondary to the initial infection will then show inhibited logarithmic bacillary growth where the infection now goes into a dormant state [13]. During the dormant/latent state,* M. tb* escapes immune surveillance via several strategies. It is capable of inhibiting both phagosome maturation and autophagy as well as translocating from the phagolysosomes to the cytosol of the AM [14, 15].* M. tb* is also capable of downregulating proinflammatory cytokines, gamma-interferon (IFN-gamma), and gamma-interferon receptor (IFN-gammaR) which is crucial T/B-cell responses [16]. Production of anti-inflammatory cytokines such as IL-10, IL-1Ra, and IL-4 and transforming growth factor beta (TGF-*β*) on the other hand antagonizes the protective T_H_1 responses by IFN-gamma and IL-1*β* as well as inducing regulatory T-cells (Tregs) [16–20].

The development of immune-surveillance escape mechanisms does not stop at the cytokine level. Processing pathways of major histocompatibility complex (MHC) class I, MHC class II, and CD1 molecule which presents antigen have also been observed to be inhibited by* M. tb*. The mechanism with which MHC class I molecule is inhibited is yet to be clearly understood but data shows the possibility of pathogen associated molecular patterns [21], especially the 19 kDa lipoprotein antigen known as LpqH from* M. tb* to inhibit phagosome maturation and thus limit the availability of antigenic peptides from* M. tb* to be presented on MHC class I molecules [22–24]. MHC class II molecules which are important for stimulating CD4^+^ T-cells in order to generate T_M_ against diseases were more clearly observed to be attenuated by the LpqH from* M. tb*. It acts as a PAMP and triggers activation of macrophages through toll like receptor (TLR) 2. However, this chronic exposure to LpqH and* M. tb* infection causes inhibition of IFN-gamma-induced regulation genes (46%), which play important roles in presentation of antigen on MHC class II molecules [25, 26]. This eventually leads to downregulation of antigen presentation and reduced activation of CD4^+^ T-cells [27, 28].

With the combination of these mechanisms,* M. tb* would normally succeed in evading the host immune system and lays dormant or slowly multiplies within the necrotic granuloma of the infected lungs (consisting of dysregulated granuloma turnover, liquefactive necrosis, and pathological scarring). The lesion formations in the granuloma are heterologously infected by* M. tuberculosis*, giving rise to the production of the CD4^+^ and CD8^+^ cells. The microenvironment of the human lesions determines the different responses caused by the* M. tb* cells depending on the location of the bacteria in the outer lesion wall [29]. The condition within these granulomas not only provides an excellent breeding ground for multiplication of* M. tb* but also serves as a spreading ground due to access to the airways of the lungs [30]. Therefore, the immunity conferred at the early stages of the infection would only serve as a vague warning sign to the patient, and in most cases many TB patients go undiagnosed until reaching active state where the immune system is already unable to retaliate against the disease. This problem is aggravated by the fact that there is no gold standard diagnostics available for latent TB: that is, about 10% of these numbers go into active TB state [5]. Although new interferon gamma release assays (IGRA) which detect* M. tb* specific antigen release of IFN-gamma are capable of producing more accurate results than the Mantoux test, there are still problems with false positive test results [31]. Therefore, current treatment of TB has poor prognosis as treatment is delayed due to the lack in diagnostics and the inability to stimulate an immunological response when TB enters the active state. This fact also strengthens the need to develop improved vaccines which would generate stronger and lasting immunological responses against TB be it for prophylaxis or therapy.

Current trends of understanding TB have also progressed as far as the involvement of other nonconventional T-cells (iNKT, CD1 restricted T-cells, *γδ* T-cells, Th17 cells, etc. [32]), presentation of peptides by HLA-E molecules [33, 34], and recognition of glycolipids/glycoproteins by CD1 restricted T-cells [35]. These have all been extensively reviewed in the cited publications and go to show that immunity generated as a result of* M. tb* infection not only confers the CD4, CD8, and B-cells immunity but also is extended towards other parts of the immunity due to its capacity to avoid immune surveillance.

## 3. Current Prophylactic TB Vaccines and Their Developments

Historically, TB vaccine has been targeted towards prophylaxis/protective immunization with Bacille de Calmette et Guérin (BCG) which was first recognized in 1931 [36, 37]. BCG, a vaccine made of live attenuated* Mycobacterium bovis* (mycobacterium strain that causes TB in cattle), is used worldwide but is now shown to be unable to protect adults with pulmonary TB and adolescents despite showing beneficial protection in children [38]. Increasing numbers of active TB is also fueled by HIV coinfection [39–43]. HIV targets the CD4^+^ T-cells which plays an important role in protection against TB as discussed in the previous chapter. Due to this coinfection, HIV compromises T-cell based immunity generated against TB, leading to accelerated reactivation of latent TB [44].

In order to tackle such problems, new candidate vaccines are emerging from around the globe. These candidate vaccines are intended to provide not only stronger immunological responses against* M. tb* but also long lasting responses which will require stimulation of memory T- and B-cell responses [38].

One of the many examples of current emerging TB vaccines is the MVA85A vaccine, which is based on Ag85A antigen expressed by modified Vaccinia Ankara virus as a subunit vaccine. Ag85A is a mycolyltransferase found in* M. tb* during its dormant stage to mediate the transesterification of diacylglycerol as acyl donors to form lipid storage bodies causing persistence of infection [45]. The MVA vaccine, which was developed as a smallpox vaccine in 1983, was first used in several animal models, which produced immunogenic responses and provides protection against BCG vaccinated animals. Safety studies conducted on BCG treated patients also showed positive results in a phase I clinical trial [46]. The vaccination protocol as of then was BCG vaccination followed by MVA85A vaccination and then fowl pox expressing Ag85A (FP85A) vaccination in a prime-boost strategy. This strategy showed a marked increase in Ag85A-specific CD8^+^ T-cells after vaccination with FP85A [47]. Although phase I studies were successful, further clinical studies in a phase IIb randomized trial showed no efficacy compared to placebo [48]. The trial recruited an extensive number of patients (2797 infants, 1399 allocated to MVA85A and 1398 allocated to placebo) in Cape Town, South Africa, but the authors only found that the vaccine was safe and generated modest immunological responses. However, efficacy was absent and further investigations are currently ongoing in order to dissect the findings.

Although the MVAg85A phase IIb failed to show improved efficacy, efforts in developing vaccines against TB should be increased and clinical trials must be designed in a manner to incorporate current understanding such as nonconventional T-cells and presentation of glycolipids/glycoproteins to CD1 restricted T-cells. This is due to the fact that, in phase IIb trial of MVAg85A, protective efficacy was shown by BCG vaccination and thus increased efficacy was not seen in such a short period. The lack of a non-BCG vaccinated group also points towards the need for better clinical trial designs [38].

Other prophylactic vaccines, which are currently in the pipeline, are listed in Table 1. One of the many interesting vaccines includes a recombinant BCG (rBCG) designed to express a lysin from* Listeria monocytogenes*, which would promote presentation of antigens delivered to the host called VPM1002 [49]. This vaccine was cleverly designed to increase the capacity of BCG vaccine to promote CD8^+^ T-cell responses. Listeriolysin acts within the infected macrophages to induce apoptosis as well as translocation of antigens into the cytoplasm, thus increasing available antigens to be presented by MHC class I molecule. VPM1002 has since concluded its phase I trial showing safety, immunogenicity, and stimulation of both B- and T-cell responses against antigens from the vaccine [50]. However, studies as such would require further investigation and investigators would need to learn from results obtained from the MV85A clinical trials.

We have so far discussed examples of non-*M. tb* vaccines which were used in vaccination against TB. The notion of immunizing with attenuated live* M. tb* was also used by several groups to design new vaccines against TB. Despite worries that the attenuated* M. tb* is a viable microorganism that has intrinsic potential risk to produce active disease, but this type of vaccine is of great potential. For obvious reasons, the use of a live attenuated* M. tb* would provide all the necessary antigens and the mycobacterium itself would act as an adjuvant as the presence of numerous types of liposaccharides is capable of triggering the innate immunity, thus increasing generation of proinflammatory cytokines such as TNF-*α*, IL-2, and IFN-gamma.

One such example is the SO2 vaccine, a mutant strain of* M. tb* MT103 that has a disrupted phoP gene [51, 52]. The phoP gene was shown to play an important role in the virulence of* M. tb* strain MT103 and this disruption reduces the potential of replication significantly [53]. The SO2 vaccine presents several key antigens from* M. tb* which have been shown to be crucial in conferring protection compared to BCG. The ESAT-6 antigen, for example, was shown to be highly immunodominant in a* M. tb* challenge study conducted in guinea pigs which was not found in BCG vaccination [54–56]. Therefore, the use of live attenuated* M. tb* as vaccine delivers a potential alternative.

Another notable vaccine is the fusion protein HyVac4 (H4), which consists of the mycobacterial antigens Ag85B and TB10.4, which is administered with the adjuvant IC31 or DDA/MPL in BCG-induced individual. H4 was shown in a preclinical study with 6- to 8-week-old female F1 crossing of inbred male C57BL/6 and female Balb/c mice to have effectively boosted and prolonged immunity induced by BCG with immune response dominated by IFN*γ*/TNF*α*/IL-2 or TNF*α*/IL2 producing CD4 T-cells [57]. Phase II clinical trials have been announced by Aeras in March 2014 and would enroll 990 adolescents.

H56 which is a fusion protein vaccine consisting of Ag85B and ESAT-6 is very similar to H4 as it also works in order to boost the immunological responses generated by BCG. In a preclinical study with BCG inoculated cynomolgus macaques, boosting with H56/IC31 resulted in efficient containment of* M. tuberculosis* infection and reduced rates of clinical disease. This was measured by clinical parameters, inflammatory markers, and improved survival of the animals compared with BCG alone [58]. H56 is developed by Statens Serum Institut (SSI) and has since collaborated with AERAS for a clinical trial. Other notable vaccines which have shown to be of great potential have been incorporated in Table 1 above.

## 4. Current Therapeutic TB Vaccines and Their Developments

Prophylactic vaccines for TB, as previously discussed, showed that only BCG was capable of providing protection but is incomplete. Due to the fact that the protection conferred is incomplete, reactivation of TB in latently infected patients poses a great risk and eradication of disease still proves challenging [59]. With the discovery of MDR, XDR, and TDR strains of* M. tb*, treatment of active TB remains as one of the top priorities, which will complement prophylactic vaccinations in order to eradicate TB. Without drugs capable of combating these* M. tb* strains, nearly 2 million of the world population are killed yearly with some patients not even receiving any treatment [60]. New avenues and combination of drugs in clinical trials showed some form of positive results like meropenem-clavulanate in combination with linezolid-containing regimens to treat XDR TB [61]. However, a new line of thought in using therapeutic vaccines has emerged with the introduction of several different candidates.

Currently there are only a handful of therapeutic vaccines that are in the development pipeline. An example of these vaccines is an inactivated whole-cell* Mycobacterium vaccae* (*M. vaccae*). This vaccine was first introduced in 1985 to boost BCG vaccinations instead of being used in treating leprosy [62, 63]. Inactivated* M. vaccae* possesses similar immunodominant antigens to* M. tb*, thus showing an improved generation of antigen-specific lymphocytes responses especially in HIV-patients with TB. Despite showing efficacy inducing T_H_1 responses, antibody responses, especially IgG against mycobacterial antigens, were also shown to escalate in these vaccinated patients [64]. When one dose of inactivated 10^9^ *M. vaccae* was administered in combination with chemotherapy treatment, significant clinical improvements were seen in non-HIV patients in a clinical trial conducted in Uganda [65]. Another trial with three doses of* M. vaccae* also showed clinical improvements in non-HIV, chemotherapy treated patients [66]. This progress however meant that treatment with chemotherapy is still crucial.

Other potential therapeutic vaccines are also in the pipeline but one similarity between the vaccines is that they augment responses generated by chemotherapy or are used to control latent infection postchemotherapy (Table 2). Therapeutic vaccines such as CSU-F36 which is a fusion of a TLR-2 agonist and ESAT-6 antigen act as a standalone vaccine which have the potential of generating cytotoxic T-cell lymphocytes responses as strong level IL-12 was generated [67]. However, this vaccination has only managed to withstand a mild dose of aerosol* M. tb* challenge.

RUTI is a new form of immunotherapy involving the use of detoxified and liposome* Mycobacterium tuberculosis* cell fragments in the vaccine regimen (Table 2). Preclinical studies have shown that RUTI treatment has the tendency to improve the chemotherapy with increased efficiency against chronic disorders caused by the* Mycobacterium tuberculosis* mouse model (C57BL/6 and DBA/2 strains) and guinea pigs [68]. This one-month vaccination strategy utilises the bactericidal effect provided by the chemotherapy to synergistically kill the active bacilli growth and suppress the inflammatory responses generated locally. The inoculation of RUTI can be implemented after the chemotherapy in order to prevent the reactivation of the latent bacilli. The intranasal administration of the RUTI vaccine in mice contributes to the reduction in the bacillus cell counts and balanced Th1/Th2/Th3 responses without toxicity. RUTI is advantageous over other TB vaccines due to the protective properties given by the specific CD8 T-cells and humoral responses induced during the treatment. This enables the immune system to fight against a broad range of antigen with antigen-specific antibody productions upon RUTI vaccination [69].

RUTI extract has successfully elicited the pronounced immune responses caused by the recombinant mycobacterial antigens [70]. Significant protection was demonstrated in the mice sera treated with RUTI regimen in SCID mice [71]. The RUTI vaccine has shown prophylactic effect as therapeutic vaccine against tuberculosis. In C57BL mouse model, the viable bacilli count was significantly reduced in both lung and spleens after 4 weeks upon vaccinations. Stronger protection was observed for lung as compared to spleen cells after 9-month vaccination. Besides, the guinea pig survived longer by giving 5-week vaccination prior to challenge. RUTI vaccination can potentially be a prophylactic treatment to reduce the risk of tuberculosis infections [72]. During phase II clinical trial, a randomised, placebo-controlled study has shown a reasonably safe vaccine which is tolerable and is immunogenic in human subjects with latent tuberculosis [69, 72].

The development of an effective TB vaccine has been challenging on the path of the licensure of the therapeutic product with proven safety and effectiveness. To design a potent prophylactic vaccine, animal models have been used extensively during vaccine development. However, successful clinical trials are required to investigate the immune response generated during an actual condition of the TB infection. Further studies are required to understand immune responses that occur naturally upon infection. To circumvent this issue, the tracking of the global TB vaccine development is important and this can be achieved by encouraging Stop TB partnership to gather vaccine pipelines to acknowledge the current candidate vaccine profile and novel therapeutic strategies. The combination of priming, booster, and therapeutic vaccines may provide protection before and after TB antigen exposure during TB infection, which can be further enhanced by rapid diagnostic approach and adjunct drug treatment against multidrug resistant TB [73, 74].

## 5. Future Directions into TB Vaccines

Current vaccinations in the pipeline are much skewed towards protective vaccination and therapy in combination with chemotherapy. This current trend is very plausible and in the light of the results obtained, be it positive or negative, it would only point towards the possible control of TB. However, we are still far away from sterile eradication of TB. In order to improve on discoveries which have already been made, we would like to underline several key factors that we think would assist in development of vaccines with enhanced efficacy.

One of the key factors that we and others think would be of crucial importance towards generating a better vaccine is the role of B-cells and antibodies [5]. Like all vaccines against infectious disease, that is, hepatitis B. virus, antibodies play the dominant role in providing initial protection against incoming infection. Antibodies are binders that not only are specific towards their targets but also recruit many arms of the immune system (NK-cells, neutrophils, and so forth) to the site of infection, thus increasing the chance of sterile eradication. Although we have discussed the ability of* M. tb* to evade this mode of eradication, we think that by introducing a vaccine capable of triggering Th2 responses and an appropriate antigen especially during the latent stage might generate antibodies which are targeted against pulmonary* M. tb*. The discovery of downregulated invariant natural killer T-cells (iNKT cells) in peripheral blood of TB patients [75] also points towards the possibility of using antibodies as a mode of targeting latent infection and activation of iNKT cells via *α*-galactosylceramide (*α*-GalCer) [32, 76] would be able to lead to the destruction of latently infected cells. However, as mentioned by Nunes-Alves et al., the use of *α*-GalCer has yet to be explored. This however can be substituted by other iNKT cells activator such as the minor lipid species that copurifies with *β*-GlcCer in mammals [77]. Aside from inducing antibody responses, the use of appropriate antigen is key to generating cytotoxic antigen-specific CD4^+^ and CD8^+^ T-cells. As we have observed in current vaccines being developed, they were mostly targeted against Ag85A, Ag85B, and ESAT-6. These targets are all very immunodominant and if so, with latent TB constantly stimulating similar T-cell clones and results showing upregulation of Tregs in patients, there will also be Tregs that are specifically targeted towards suppressing T-cell responses against these antigens. This situation is often noticed in chronic diseases as well as in cancer whereby antigen-specific Tregs are often found to have superior suppressive capacity, thus leading to suppression of T-cell responses [78]. We speculate that this might be one of the reasons why results obtained from clinical trials are inferior compared to results obtained from animal studies. Generating T-cell responses against* M. tb* which is yet to be suppressed would require the search of new antigens which are constitutively expressed yet less immunodominant. One example of such possible antigen is Mtb32 that, despite being less studied, has shown to be a promising antigen for generating CD4^+^ and CD8^+^ T-cell responses in pre- and postexposure in* M. tb* mouse models [79, 80].

Another key factor in designing a potential vaccine would be the use of an adjuvant which would provide depot effects, stimulate the innate immunity to provide a proinflammatory environment, and skew Th responses [81]. A combination of a Squalene/Tween-80 emulsion, 2 TLR agonist, an MHC class I target peptide, an MHC class II helper peptide, and IFN-gamma has recently showed generation of high levels of antigen-specific CD8^+^ T-cells which not only are cytotoxic but also were shown to generate memory T-cells. Responses generated by this combination (CASAC adjuvant) not only were against foreign antigens (ovalbumin) but also were capable of mounting responses towards self-antigen (tyrosinase-related protein 2). Responses generated were capable of eradicating a B16 mouse melanoma challenge. The responses generated could also be recalled after a 50-day resting period [82].

One final key factor that we should factor in is to use antibodies that would deplete or block suppressive signals such as programmed-death 1 (PD-1), cytotoxic T-lymphocyte antigen 4 (CTLA-4), and ultimately Tregs. PD-1, CTLA-4, and Tregs are all correlated in suppressing T-cell responses. In a recent study to develop cancer vaccines, PD-1 and CTLA-4 have shown to be present on tumour infiltrating lymphocytes (TIL), antigen-specific CD8^+^ T-cells. This presence was regulated by Tregs which at the end causes dysfunction of cytotoxic T-cells that were infiltrating tumours. In a comparative study, dual blockade of PD-1 and CTLA-4 restored the dysfunction of these TIL and caused 100% rejection of tumour [83]. This could be a very interesting avenue to pursue in order to possibly restore functions of reactive T-cells that might have been generated in the initial infection stage of TB as described previously.

Based on these lines of thoughts, we suggest the possibility of developing new vaccines based on the use of an adjuvant such as CASAC in combination with new antigens which is of less immunodominance and pretreatment with antibody that depletes or blocks suppressive responses such as anti-PD-1 and anti-CTLA-4.

On a similar train of thoughts, we think that using heat shock proteins (HSP) as an immunogenic carrier could be of potential benefit [84–86]. HSP are a group of proteins that is recognized by the human immune system and expressed during inflammation. The human immune system has a natural autoimmunity towards HSP, particularly HSP60 here, whereby CD4^+^ T-cells responses would be generated. HSP60 showed positive preclinical results in multiple occasions as an adjuvant which will provide help towards both CD8^+^ T-cells and B-cells due to activation of the CD4^+^ T helper cell in diseases such as murine CMV [85], Meningitides [87], and West Nile virus [88]. Therefore, with the right selection of* M. tb* antigen or attenuated whole* M. tb* cell in combination with potent adjuvant and the right pretreatment, we think that generation of a new line of vaccine for TB would not be a far cry away in the hope of eliminating TB.

## Figures and Tables

**Table 1:** Candidate vaccine with emerging protective responses and its brief description.

TB vaccine	Vaccine details
MVA85A [45]	Modified Vaccinia Ankara virus expressing Ag85A

VPM1002 [50]	rBCG expressing listeriolysin

AdAg85A [89]	Adenovirus expressing Ag85A

ΔIKEPLUS [90]	*Mycobacterium smegmatis* mutant that expresses *M. tb* esx-3 genes which is capable of inducing central memory responses

SO2 [52]	Mutant *M. tb* strain that has phoP deleted

Hybrid 1 + IC31 [91]	Fusion of ESAT-6 and Ag85B in adjuvant IC31

H4 [57]	Ag85B and TB10.4 administered with IC31 or DDA/MPL

H56 [58]	Ag85B and ESAT-6 administered with IC31

rBCG30 [92, 93]	30-kDa major secretory protein of *Mycobacterium tuberculosis *

M72 + AS01/AS02 [94]	Fusion protein of Rv1196 and Rv0125 in AS01 or AS02 adjuvant

**Table 2:** Candidate emerging therapeutic TB vaccines and its brief description.

Vaccine	Vaccine details
RUTI [68]	A liposome that encapsulates detoxified *M. tb* fragments

*Mycobacterium indicus pranii* (MIP) [95]	A live saprophytic mycobacterium administered via aerosol route

CSU-F36 [67]	A fusion of Rv1411 (TLR-2 agonist) and ESAT-6 protein
